# A proposition for a new term: "Rhonchoplastic Injection"

**DOI:** 10.1016/S1808-8694(15)30562-0

**Published:** 2015-10-19

**Authors:** Fábio Tadeu Moura Lorenzetti, Gilberto Guanaes Simões Formigoni, Michel Burihan Cahali

**Affiliations:** 1 Specialist by the Associação Brasileira de Doutor em Ciências, Medical School of the Universidade de Sao Paulo (FMUSP). Clinical supervisor of the Otorrinolaringologia Unit of the Hospital das Clínicas, FMUSP.; 2 Otorhinolaryngology and Cervicofacial Surgery. Title of Sleep Medicine, Associação Brasileira de Sono. Doctoral student in Otorhinolaryngology, Faculdade de Medicina (Medical School), Universidade de São Paulo (FMUSP).; 3 Doctor in Sciences, Faculdade de Medicina (Medical School), Universidade de São Paulo (FMUSP). Collaborating professor, FMUSP, and assistant physicians in the Hospital das Clínicas, FMUSP. Hospital das Clínicas, Faculdade de Medicina (Medical School) da Universidade de São Paulo.

**Keywords:** obstructive sleep apnea, sclerotherapy, snoring, therapy

Dear Editor,

One of the themes that has arisen frequently in recent ENT conferences is soft palate sclerotherapy for the treatment of snoring and sleep disordered breathing (SDB); this method is a promising option for outpatient therapy, given its ease and low cost. Although this theme is discussed routinely, its naming lacks standardization. Our proposition is to suggest a new term in Portuguese for this form of treatment: “Injeção Roncoplástica” (Rhonchoplastic Injection).

In medical practice, the term sclerotherapy is well established in naming the esthetic treatment of varices and telangiectasis; it may be used to describe numerous and distinct types of treatment, such as in the approach to thyroid cysts, hydroceles, cystic hygroma, hemangiomas, venous malformations, esophageal varicose veins, hemorrhoids, and others.

Even when this theme is discussed in papers and otorhinolaryngological conferences, there is no standard name for the use of sclerosing agents in the treatment of snoring, which might lead to confusion. The same theme has been described as: sclerotherapy for the treatment of snoring, injection of sclerosing substances for the treatment of snoring, soft palate treatment with sclerosing substances, interstitial therapy for snoring, etc.

Although Straus (1943) was the first to use sclerotherapy for the treatment of snoring, Brietzke and Mair were truly responsible for the main studies of this treament.^1^, ^2^, ^3^ These authors published papers in 2001 and 2003 describing the use of Sotradecol® (sodium tetradecyl sulphate) injected into the human soft palate, with excellent clinical results for the treatment of snoring: a 75 to 92% success rate after a 12 to 19 month follow-up.^1^, ^2^ In 2004, Brietzke and Mair published a comparative study using various types of sclerosing substances for the treatment of snoring. The found that 50% ethanol was similar in efficacy to Sotradecol®, albeit with a slightly higher complication rate.^3^ Few papers have been published on sclerotherapy for the treatment of snoring to date, but those that have been published report favorable results.^1^, ^2^, ^3^, ^4^, ^5^, ^6^

There are very few studies on this theme in Brazil. In 2002, Lopes et al. injected Ethamolin® (monoethanolamine oleate) in the base of the tongue of pigs and then analyzed histologically the injection sites. They found that collagen had become compacted and thickened and that fibrosis had formed much more significantly compared to controls.^6^ We are currently running a study on human soft palate sclerotherapy for the treatment of snoring and SDB. In this study we compare 50% ethanol with Ethamolin® injections. Partial results have been forthcoming and we hope to publish these findings shortly.

The term sclerotherapy is well established and widely disseminated for naming many different medical therapies, especially sclerosis of varicose veins and telangiectasis. Thus, our proposition is to use the term “Injeção Roncoplástica” (Rhonchoplastic Injection) in Portuguese specifically to designate the injection of sclerosing substances into the soft palate for the treatment of snoring and SDB. We believe that this term is more adequate, since it is distinct and self-explanatory, and closer to the English term “Injection Snoreplasty”.
